# Epidemiology of pediatric burns and future prevention strategies—a study of 475 patients from a high-volume burn center in North India

**DOI:** 10.1186/s41038-016-0067-3

**Published:** 2017-02-01

**Authors:** Amol Dhopte, V. K. Tiwari, Pankaj Patel, Rahul Bamal

**Affiliations:** 1 Department of Burns, Plastic & Maxillofacial Surgery, VMMC & Safdarjung Hospital, New Delhi, India; 2 Present Address: Department of Burns & Plastic Surgery, PGIMER & RML Hospital, New Delhi, India

**Keywords:** Epidemiology, Pediatric burns, India, Suicidal burns, Burn prevention

## Abstract

**Background:**

Pediatric burns have a long-term social impact. This is more apparent in a developing country such as India, where their incidence and morbidity are high. The aim of this study was to provide recent prospective epidemiological data on pediatric burns in India and to suggest future preventive strategies.

**Methods:**

Children up to 18 years old admitted to the Department of Burns, Plastic & Maxillofacial Surgery, VMMC & Safdarjung Hospital, New Delhi, between January and December 2014 were included in the study. Data regarding age, sex, etiology, total body surface area (TBSA), circumstances of injury, and clinical assessment were collected. The Mann-Whitney test or Kruskal-Wallis test or ANOVA was used to compare involved TBSA among various cohort groups accordingly. Univariate and multivariate linear regression analyses were used to determine the predictors of TBSA.

**Results:**

There were a total of 475 patients involved in the study, including seven suicidal burns, all of whom were females with a mean age greater than the cohort average. Age, type of burns, mode of injury, presence or absence of inhalation injury, gender, and time of year (quarter) for admission were found to independently affect the TBSA involved. Electrical burns also formed an important number of presenting burn patients, mainly involving teenagers. Several societal issues have come forth, e.g., child marriage, child labor, and likely psychological problems among female children as suggested by a high incidence of suicidal burns.

**Conclusions:**

This study also highlights several issues such as overcrowding, lack of awareness, dangerous cooking practices, and improper use of kerosene oil. There is an emergent need to recognize the problems, formulate strategies, spread awareness, and ban or replace hazardous substances responsible for most burn accidents.

## Background

Pediatric burns can have long-term physical, psychological, economic, and social implications for patients and their families with ongoing treatment, rehabilitation, and the need for regular interventions. Pediatric burns occur more frequently in developing countries, with their incidence being many times higher in low- and moderate-income countries compared to high-income countries [1, 2].

Studies worldwide have demonstrated that the incidence of burn injuries is highest among children below 4 years of age, with responsible factors ranging from children’s impulsiveness, lack of awareness, higher activity levels due to natural curiosity, and total dependency on caregivers [3–11].

Pediatric burns are also known to occur due to several other factors, including lack of proper supervision, use of common areas for both cooking and sleeping, traditional habits of cooking over low stoves or in large pots (cheese making), consuming food while sitting on the floor, transferring hot liquids in open containers from one place to another, and sterilization of milk by boiling rather than pasteurization [12–15].

Electrical burns in children are generally caused by domestic electric current, which is 220–250 V in India. Children often bite electrical cord, sustaining a burn of the lip. Alternatively, children may introduce a finger or an object such as a metallic hairpin into a power socket, thus suffering an electrical injury. Firecracker injuries are also a common cause of burn and hand injuries in North India, especially during the festival season of Diwali [16, 17].

Epidemiological data on pediatric burns can provide vital information for developing prevention strategies, thus reducing the frequency of such burns and the budgetary demands on the health care system. Such studies from India are few, and most of them have a retrospective design. Furthermore, significantly less data were analyzed and reported by these studies. We do not have recent and reliable documentation of the exact magnitude of burn injuries among children from India. Such data can encourage the government to take initiatives to prevent this menace [1, 8, 18, 19].

The aim of this study was to provide recent prospective epidemiological data on pediatric burns in India, defining important etiologies such as suicidal burns and suggesting future high impact preventive strategies.

## Methods

Subjects were patients up to 18 years old admitted to the Department of Burns, Plastic & Maxillofacial Surgery, VMMC & Safdarjung Hospital (SJH), New Delhi, between January and December 2014. SJH is one of the largest tertiary care burn centers in the country. It receives patients mainly from Delhi and neighboring states. Ours is a dedicated burn unit managed by plastic surgeons. We have a 12-bed Burn Intensive Care Unit (BICU) with three reserve beds, a 17-bed step-down burn ward and a 32-bed general burn ward. We also have a dedicated burn operating theater and a physiotherapy unit.

Registration data, data on circumstances of the injury and clinical assessment were collected from all patients for this observational, analytical, and prospective assessment. Questions were asked by resident doctors admitting the patients. Data were collected during the next 24 h of admission from the clinical notes after confirmation from the index person (doctor) writing the notes if required. Either the attendant or patient answered all the questions depending on the age and the condition of the patient.

Standard Lund and Browder charts as appropriate for patient age were used for rapid assessment of total body surface area (TBSA) involved.

Clinical clues of inhalation injury included suggestive history, increased respiratory rate, hoarseness, altered mental status, head and neck burns, singed nasal hairs, inflamed oral mucosa, and carbonaceous sputum.

The Mann-Whitney test was used to compare TBSA between males and females, inhalation injury (yes/no), and type of referral (direct/referred). The Kruskal-Wallis test was used for comparison of TBSA between age groups, time of year (quarter), and type of burn. ANOVA was used to compare TBSA between different modes of injury.

Univariate and multivariate linear regression analyses were used to determine the predictors of TBSA. Univariate linear regression analysis was performed using TBSA as the dependent variable, with age, burn type, mode of injury, gender, inhalation injury, time of year (quarter), and type of referral as independent variables. Multivariate linear regression analysis was then performed for significant independent variables. The linear regression *F* test was used to analyze the linear relationships among the variables.

## Results

### Cohort characteristics

**Fig. 1:**
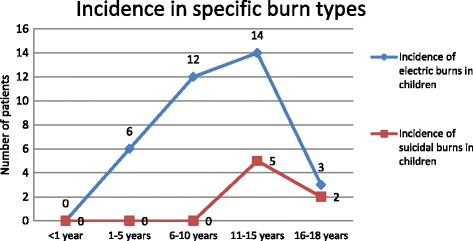
Incidence of specific burns (electric and suicidal burns) in children by age group

A total of 475 pediatric patients were hospitalized at our center during the assessment period. The mean age of patients was 6.52 ± 5.4 years with 238 (50.1%) patients between 1 and 5 years of age (Table 1). The incidence of electrical burns (*n* = 14) as well as suicidal burns (*n* = 5) in 11–15-year-old children depicted an upward trend compared to younger age groups, where there were 12 and 6 electrical burn patients in the 6–10-year and 1–5-year age groups, respectively. Additionally, there were no suicidal burn cases in the younger age groups (Fig. 1). There were 281 males and 194 females, giving a male to female (M:F) ratio of 1.45:1. The mean TBSA involved increased from 25 ± 15% to 59 ± 24% with increasing age from <1-year to 16–18-year-old. This association was found to be statistically significant. Similarly, mean TBSA was significantly higher in females 43 ± 28% compared to males 33 ± 20% (Table 1).

**Table 1:** Correlation of multiple variants with total burn surface area (TBSA); (SD = standard deviation)

Variables	Number of cases (*n*)	Cases in percentage (%)	Mean TBSA ± SD (%)	*P* value
Age group (mean±SD age = 6.52 ± 5.4 years)				
<1 year	28	5.9	25 ± 15	<0.0001
1–5 years	238	50.1	29 ± 17	
6–10 years	78	16.4	33 ± 19	
11–15 years	91	19.2	56 ± 28	
16–18 years	40	8.4	59 ± 24	
Type of burn				<0.0001
Scalds	232	48.8	27 ± 15	
Thermal	206	43.4	48 ± 27	
Electric	34	7.2	36 ± 24	
Chemical	3	0.6	28 ± 13	
Mode of injury				<0.001
Accidental	463	97.5	36 ± 23	
Homicidal	2	0.4	62 ± 52	
Suicidal	7	1.5	84 ± 22	
Not specified	3	0.6	72 ± 14	
Inhalational injury				<0.0005
Yes	74	15.6	63 ± 26	
No	401	84.4	32 ± 20	
Gender				0.001
Male	281	59.2	33 ± 20	
Female	194	40.8	43 ± 28	
Time of year (quarter)				<0.0001
Jan–Mar	137	28.8	32 ± 21	
Apr–Jun	114	24	45 ± 27	
Jul–Sep	124	26.1	40 ± 25	
Oct–Dec	100	21.1	30 ± 18	
Type of referral				0.004
Direct	250	52.6	35 ± 25	
Indirect	225	47.4	39 ± 23	
Total	475	100	37 ± 24

A total of 460 (96.8%) patients were either students or unemployed, while a few patients worked as laborers (*n* = 3), cooks (*n* = 2), farmers (*n* = 2), garbage collectors (rag pickers) (*n* = 2), and photographer (*n*=1). Four female patients were married and worked as housewives, while one patient was running a shop. Most burns [*n* = 416 (87.6%)] were sustained inside the home, either in the kitchen, living room, or bathroom. Most of the patients belonged to nuclear families [*n* = 381 (80.2%)], and 299 (62.9 %) patients had a family size of fewer than six members (Table 2).

**Table 2:** Table showing various cohort characteristics as mentioned

Variables		Number of cases (*n*)	Cases in percentage (%)
Occupation	Nil	341	71.8
	Cook	2	0.4
	Farmer	2	0.4
	Garbage collector (rag pickers)	2	0.4
	Housewife	4	0.8
	Laborer	3	0.6
	Photographer	1	0.2
	Shopkeeper	1	0.2
	Student	119	25.1
Family size (members)	1–5	299	62.9
	6–10	161	33.9
	>10	15	3.2
Place of sustaining burns	Kitchen	250	52.6
	Living room	138	29.1
	Bathroom	28	5.9
	Out of house	59	12.4
Family type	Joint	94	19.8
	Nuclear	381	80.2
Depth of burns	Full	41	8.6
	Mixed	199	41.9
	Partial	235	49.5
Kitchen type	Floor	293	61.7
	Standing	181	38.1
	Tent	1	0.2
Residential locality	Rural	143	30.1
	Urban	332	69.9

### Burn characteristics

**Fig. 2:**
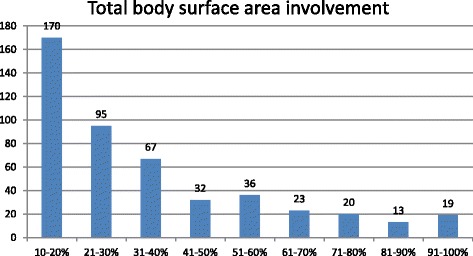
Number of patients represented graphically according to TBSA (percentage of TBSA involved with burns) in different groups

In total, 232 (48.8%) patients sustained scalds, while 206 (43.4%) had thermal burns. Additionally, 463 (97.5%) cases sustained accidental injuries, while seven (1.5%) injuries were suicidal in nature. All suicidal burn victims were female children. The overall mean TBSA involved in our study was 37 ± 24% against an average of 84 ± 22% when only the cases of suicidal burns were considered (Fig. 2). Additionally, all suicide victims used an inflammable liquid such as kerosene oil to commit suicide. There were also two patients with homicidal burns in our study. In 74 (15.6%) patients with inhalation injury, the mean TBSA involved was 63 ± 26%, with 5 out of 7 suicidal burns having inhalation injuries (Table 1).

### Temporal variation and type of referral

The number of admissions during different quarters of the year varied from 137 (28.8%) patients between January and March to 100 (21.1%) patients between October and December. A total of 250 (52.6%) patients came directly to us, while 225 (47.4%) were referred from other hospitals (Table 1).

### Depth of burns and pre-hospital first aid

**Fig. 3:**
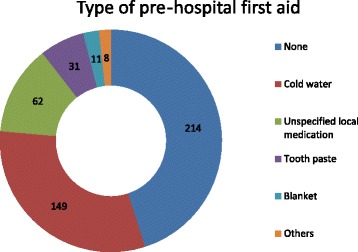
Depiction of varying types of pre-hospital first aid provided to patients

The highest proportion of patients (49.5%) sustained partial thickness burns, while 199 (41.9%) patients had mixed thickness burns (Table 2). Regarding pre-hospital first aid, only 261 patients received some form of first aid. Among these, cold water was used in most cases (*n* = 149). Other methods varied from unspecified medicinal creams (*n* = 62) to toothpaste (*n*=31), blankets (*n*=11), Ratan Jot, ice, ink, potato peel, and sand (1 patient each) in other cases (Fig. 3).

### Statistical analysis

After performing univariate analysis, patient age, type of burns, mode of injury, presence or absence of inhalation injury, gender, and time of year (quarter) of admission were found to be independent predictors of TBSA (Table 3).

**Table 3:** Univariate linear regression with TBSA as dependent variable

Variables	Unstandardized coefficients		Standardized coefficients	*P* value	95.0% confidence interval for B	
	B	Std. error	B		Lower bound	Upper bound
Age	0.022	0.002	0.498	<0.0005	0.018	0.025
Type of burn (scald as reference)	0.274	0.014		<0.005	0.246	0.302
Thermal	0.206	0.021	0.430	<0.005	0.165	0.246
Chemical	0.010	0.126	0.003	0.940	−0.238	0.257
Electric	0.090	0.040	0.098	0.023	0.012	0.168
Mode of injury (accidental as reference)	0.359	0.011		<0.0005	0.338	0.380
Homicidal	0.256	0.162	0.070	0.115	−0.062	0.574
Not specified	0.358	0.132	0.120	0.007	0.098	0.618
Suicidal	0.481	0.087	0.245	<0.0005	0.310	0.652
Gender (male as reference)	0.098	0.022	0.204	<0.0005	0.056	0.141
Inhalation injury (“no” as reference)	0.314	0.026	0.481	<0.0005	0.262	0.366
Time of year (quarter) (Oct–Dec as reference)	0.302	0.023		<0.0005	0.257	0.348
Jan–Mar	0.015	0.030	0.028	0.626	−0.045	0.074
Apr–Jun	0.151	0.032	0.272	<0.0005	0.089	0.213
Jul–Sep	0.102	0.031	0.188	0.001	0.041	0.162
Type of referral (direct as reference)	0.038	0.022	0.08	0.081	−0.005	0.081

Multivariate analysis revealed that higher age, suicidal mode of injury, presence of inhalational injury, and admissions between April and September were significantly associated with a higher percentage of TBSA involvement (Table 4).

**Table 4:** Multivariate linear regression with TBSA as dependent variable

Variables	Unstandardized coefficients		Standardized coefficients	*P* value	95.0% confidence interval for B	
	B	Std. error	B		Lower bound	Upper bound
Age	0.014	0.002	0.317	<0.005	0.010	0.018
Type of burn (scald as reference)						
Thermal	0.039	0.023	0.082	0.085	−0.005	0.084
Electric	−0.029	0.038	−0.031	0.448	−0.103	0.045
Mode of injury (taking accidental as reference)						
Not specified	0.130	0.111	0.043	0.244	−0.089	0.348
Suicidal	0.198	0.073	0.100	0.007	0.053	0.342
Gender (male as reference)	0.031	0.018	0.064	0.092	−0.005	0.067
Inhalation injury (“no” as reference)	0.185	0.027	0.283	<0.005	0.132	0.238
Time of year (quarter) (Oct–Dec as reference)						
Apr–Jun	0.065	0.022	0.116	0.004	0.021	0.108
Jul–Sep	0.045	0.021	0.083	0.035	0.003	0.087

We found that the adjusted *R* ^2^ of our model is 0.389. This means that linear regression explains 38.9% of the variance in the data. Furthermore, the Durbin-Watson *d* = 1.883 (between the two critical values of 1.5 < *d* < 2.5) indicates that there is no first-order linear autocorrelation in our multiple linear regression data.

Beta expresses the relative importance of each independent variable in standardized terms.

First, we found that age, suicidal mode of injury, inhalation injury, and time of year (quarter) were significant predictors. Second, we found that age had the greatest impact (beta = 0.317), followed by other variables. Multicollinearity in our multiple linear regression model was checked, with tolerance being >0.1 (or VIF <10) for all variables (Table 4).

## Discussion

This study was designed with the aim of providing recent prospective epidemiological data on pediatric burns in India. Stress was placed on important etiologies such as suicidal burns and suggesting future high-impact preventive strategies.

The admission of 475 pediatric patients in a single year is probably the most for any center in the country, reflecting the high incidence of burn-related injuries in this part of India. Other similar studies from India are retrospective in nature, whereas we have presented prospective data. This study presents an exceedingly high volume of patients in a single year compared to earlier literature, which studied no more than 100 patients in the same period. Data in already published studies were reported predominantly from other parts of India, while our study provides recent data from North India. Most other studies included children below 14 years of age. However, we designed a more comprehensive study including all patients below 18 years of age. Other studies were limited in their explanations of epidemiological profiles. Furthermore, they did not use any statistical analyses to show the strengths of associations of different factors. No previous study from North India mentioned homicidal and suicidal burns in children, type and size of families of pediatric burn patients, the area of victims’ residence (rural or urban), and depth of burns. We provided a detailed profile of our patients by incorporating all of the points mentioned above and used statistical tools efficiently. Our novel findings included suicidal burns among female children and the different occupations in which patients were involved [1, 8, 18–20].

Burn care in India is not very well organized. There are no official data on either the number or distribution of dedicated burn care beds available in the country. The number can range anywhere from 500 to a few thousand in government sector hospitals. The government sector handles most of the burn care load. The referral of these cases from the periphery depends on the treating doctor, extent of burns in patients, capacity of the center, and distance from a specialized center. We can broadly classify burn care stratification in India into general beds, dedicated general surgery beds for burn patients, dedicated burn care beds in burn units managed by general surgery and dedicated burn units managed by plastic surgery.

Consistent with the literature, children between 1 and 5 years of age in our study were shown to be at the greatest risk, thus making them the prime targets for prevention. Males were affected more than females, similar to reports from previous studies. This may be attributable to the mischievous nature and greater activity levels of boys. On the contrary, all suicidal burn patients were females, warranting consideration that might point to the prevalence of poor mental well-being among female children [3–10]. Scalds (48.8%) were predominant among our patients, closely followed by thermal burns (43.4%). These data were similar to data from other studies. However, some studies reported a higher incidence of thermal burns [1, 4, 8, 15, 21, 22].

Older children were more likely to be burned by flames. This finding is supported by an Australian study that reported that 95% of flammable liquid burns occurred in young adolescent males. Sometimes while playing, children’s clothes may accidentally catch fire. Adolescents and teenagers with improved social interaction and higher emotional quotients tend to commit suicide by pouring kerosene oil and lighting themselves ablaze due to various psychological issues. We identified seven such girl children. There were also two cases of homicidal burns in our study. On multivariate regression analysis, the suicidal mode of injury was found to be significantly associated with a higher involved TBSA. Most suicide victims (*n* = 5) had more than 95% of TBSA involved. Another recent study of 122 pediatric patients also identified 7 cases of suicidal burns and one homicidal burn case during their 5-year study period [1, 23, 24].

In our study, 34 (7.5%) patients sustained burns due to electrical injuries. Three patients suffered chemical burns caused by corrosive substances. Similarly, another study from a tertiary care children’s hospital in India reported 38 pediatric patients with chemical burns during a study period of 10 years [16].

Admissions were higher in the first and third quarters of the year, while TBSA involved was significantly higher among patients admitted between April and September 2014. This finding may be attributable to hot weather. Studies have reported variations in burn incidence with changing weather and during festivities with the use of firecrackers. However, in our study, there was no such increase in the last quarter, during which the festival of Diwali is celebrated. This trend can be attributed to vigilance by elders and awareness and management of most firecracker burns on an outpatient basis [1, 18, 21, 25, 26].

Studies have reported that the majority of pediatric patients sustained burns involving less than 30% TBSA. We had 55.8% patients with burns involving 10–30% TBSA. On multivariate regression analysis, older patients had significantly greater TBSA involvement [18, 19].

Inhalation injury was mainly associated with thermal injuries occurring in closed places. These patients had significantly higher TBSA involvement. Causative factors for thermal burns were mainly liquid petroleum gas (LPG) leakage in closed spaces, with the involvement of face and upper trunk and neck areas. Twelve children were found to be employed at school-going age, showing that the problem of child labor persists in the society. Three female patients were married as housewives, indicating that the stigmata of child marriage still prevail in some parts of India.

Most patients in our study belonged to nuclear families. This finding might be attributed to the fact that children from smaller families are at increased risk of sustaining burn injuries, as parental supervision cannot always be provided and fewer hands are available to care for children. This trend tends to occur as both parents work and other responsible non-working members such as grandparents might not be present to look after children. Therefore, children are taken care of by maids or young siblings, resulting in more accidents. This tendency was especially highlighted in the case of electrical burns, where 33 out of 35 patients were from nuclear families. A higher percentage of nuclear families in urban areas can be the reason for most patients belonging to these areas compared to only 30% of patients from rural areas. Few studies highlight this contrast. Nevertheless, the possibility of under-reporting of cases from rural areas should also be considered before drawing any conclusion. A review of the literature of retrospective studies on pediatric burns from China reported a higher incidence of pediatric burn injuries in the rural population, in contrast to our study [25].

The overwhelming majority of burn accidents occurred indoors (87.6%). This finding can likely be the result of children, especially younger children, living and playing mainly indoors. Studies have also revealed that indoor burns occurred primarily in the kitchen and bathroom. Our findings suggest that burn prevention strategies should focus on indoor burns, as that was the location where most burns were sustained in this age group [12, 27].

We found that 61.7% of accidents occurred in households that use the floor for cooking. These data are very typical of rural India but also occur in urban areas among people belonging to low socio-economic strata. This finding adds substantially to the risk of burn-related injuries. Young children playing on the floor tend to reach toward whatever is being cooked in the kitchen. As a result, they may sustain burn injuries. Due to a lack of supervision, children may pull on kitchen utensils lying overhead or they may fall accidentally into containers and stay there until someone rescues them.

Most thermal burns seen in our study were mainly due to LPG gas leakage. We found that the falling over of kerosene oil lamps from a height, electric switches causing sparks after LPG leaks in kitchens, and improper operating knowledge of LPG gas cylinders were important causes of many burn accidents. Due to overcrowding and sharing of kitchens and living rooms, fire due to these sources also tends to affect children as well as other family members.

We noted that the percentages of patients reporting directly to our casualty and those referred from other centers were nearly equal. These data reflect the public awareness of the availability of tertiary care centers in their region.

Standard worldwide guidelines consist of providing first aid by running cool water for 20 min. Cooling blankets are also suggested; however, cost and availability are limiting factors. We should also take care to avoid hypothermia, especially in children. Our study identified poor societal knowledge with regard to first aid for a burned patient. Only 57% of patients were provided with cold water as a first aid measure. In other cases, either no first aid was provided or it included medicinal creams, ice, ink, potato, Ratan Jot (alkanet), and toothpaste. We need to improve society’s knowledge in this area.

Full-thickness burns were noted in only 8.6% of patients, which was fewer than the 15% of pediatric patients identified with III or IV degree burns in a recent study of 122 patients over 5 years. Full-thickness burns occur when the child cannot be rescued in time, resulting in a longer period of contact with the causative agent [24].

A major limitation of this study was the likely inability to accurately quantify the incidence and risk factors for pediatric burns, as this was a referral hospital-based study. Additionally, patients who did not need admission were not included in the study. Medical personnel would have performed triage at different points of referral and might have transferred only selected patients based on different guidelines or protocols. Outcomes consisting of morbidity and mortality data are not reported, as our aim was to focus only on epidemiology while reporting these data. Future studies along with outcomes will be helpful.

## Conclusions

The most important group of pediatric burns that should be the prime target of prevention is among those aged between 1 and 5 years. The kitchen, bathroom, and living room are common areas of accidents, where proper precautionary measures should be undertaken. The age of the patient, type of burns, mode of injury, presence or absence of inhalation injury, gender, and time of year (quarter) of admission were found to be factors independently affecting the involved TBSA.

This study highlights poor community knowledge, as evidenced by the approach toward first aid of burn patients. There is a need to formulate measures to prevent burn-related accidents in children through education of parents and guardians. Proper surveillance of the children should be emphasized. We have outlined various risk factors for pediatric burns in our setup through this study. This framework should aid the formulation of effective burn prevention programs in this country as well as the development of prevention strategies. Kerosene oil is one of the main causes of thermal burns in the form of lit oil lamps placed inappropriately as well as its role in adolescent suicidal burns. Kerosene oil should be phased out from households throughout the country, as has been done in the national capital since 2012. We propose that the unregulated and illegal sale of LPG should be strictly prohibited in the country. The entire country should switch to safer piped cooking gas (PNG-piped natural gas), as is being done in various major cities. There should be awareness about proper usage of LPG, with specific emphasis on not using electric switches in the event of suspected leakage. It should be mandatory to have electrical switches located outside rather than inside the kitchen.

Moreover, we want to stress that older children should be taught and supervised to reduce the currently high incidence of electrical burns in this age group. There should be no exposed wires or unguarded sockets within reach of children. A high incidence of suicidal burns among adolescent children, especially females, was noted. There should be better communication between children and parents, as this will help address their psychological issues at an earlier stage. Parents should be more understanding and open to their children’s perspectives and issues at the same time.
